# High-Precision Visual Servoing for the Neutron Diffractometer STRESS-SPEC at MLZ

**DOI:** 10.3390/s24092703

**Published:** 2024-04-24

**Authors:** Martin Landesberger, Oguz Kedilioglu, Lijiu Wang, Weimin Gan, Joana Rebelo Kornmeier, Sebastian Reitelshöfer, Jörg Franke, Michael Hofmann

**Affiliations:** 1 Heinz Maier-Leibnitz Zentrum (MLZ), Technical University of Munich (TUM), 85748 Garching, Germany; lijiu.wang@frm2.tum.de (L.W.); joana.kornmeier@frm2.tum.de (J.R.K.); michael.hofmann@frm2.tum.de (M.H.); 2 Faculty of Engineering and Management, Technische Hochschule Ingolstadt, 85049 Ingolstadt, Germany; 3 Institute for Factory Automation and Production Systems (FAPS), Friedrich-Alexander-Universität Erlangen-Nürnberg (FAU), 91058 Erlangen, Germany; oguz.kedilioglu@faps.fau.de (O.K.); sebastian.reitelshoefer@faps.fau.de (S.R.); joerg.franke@faps.fau.de (J.F.); 4 Helmholtz-Zentrum Hereon, 21502 Geesthacht, Germany; weimin.gan@hereon.de

**Keywords:** neutron diffraction, computer vision, robotics, high accuracy, residual stress analysis, visual servoing, automation, texture analysis

## Abstract

With neutron diffraction, the local stress and texture of metallic components can be analyzed non-destructively. For both, highly accurate positioning of the sample is essential, requiring the measurement at the same sample location from different directions. Current sample-positioning systems in neutron diffraction instruments combine XYZ tables and Eulerian cradles to enable the accurate six-degree-of-freedom (6DoF) handling of samples. However, these systems are not flexible enough. The choice of the rotation center and their range of motion are limited. Industrial six-axis robots have the necessary flexibility, but they lack the required absolute accuracy. This paper proposes a visual servoing system consisting of an industrial six-axis robot enhanced with a high-precision multi-camera tracking system. Its goal is to achieve an absolute positioning accuracy of better than 50μm. A digital twin integrates various data sources from the instrument and the sample in order to enable a fully automatic measurement procedure. This system is also highly relevant for other kinds of processes that require the accurate and flexible handling of objects and tools, e.g., robotic surgery or industrial printing on 3D surfaces.

## 1. Introduction

Neutron diffraction has proven to be a state-of-the-art non-destructive method for stress and texture analysis in metallic components [1]. Current high-accuracy sample-positioning systems at in neutron diffraction instruments combine three linear axes (XYZ tables) and three rotation systems (usually Eulerian cradles) to cover the 6DoF sample manipulation. These systems provide an accuracy below 5 μm and, in the case of Stewart platforms, less than 1 μm [2]. However, in the case of conventional systems, a free choice of the sample rotation center is not available, while in the case of Stewart platforms, tilt angle coverage is severely limited. Large industrial six-axis robots can overcome this drawback with their flexibility and freedom of rotation center choice, but they often lack the necessary absolute positioning accuracy. Therefore, robotic systems at large-scale material science facilities are currently used only for sample exchange and positioning tasks, such as texture measurement, where high accuracy is not mandatory. Altenkirch et al. were the first to recognize the potential of a robotic sample manipulation system for neutron strain scanning [3]. However, the first real installment at a neutron beamline was initiated by Brokmeier et al. [4,5] at the materials science diffractometer STRESS-SPEC at the research reactor FRM II. Since 2010, the heavy load robot at STRESS-SPEC has been in full user operation as the main option for texture measurements at the diffractometer and occasionally for strain determination in larger objects [6]. A similar system was implemented later also by [7] at the HIPPO instrument of the spallation neutron source LANSCE. They integrated a Staubli TX40 robot in the instrument control environment. That particular robot checks for collisions through motor force surveillance and is used mainly for sample exchange and texture measurements. Similar robot arrangements can be found in the KOWARI instrument at ANSTO [8]; the RSND diffractometer in Mianyang, China [9]; and the HK4 strain scanner at the Nuclear Physics Institute Rez, Czech Republic [10]. A robot positioning system is planned at the ESS engineering diffractometer BEER [11], and robots will also be used for detector alignment at IMAT (ISIS) [12] and the single-crystal diffractometer NMX of the European spallation source ESS in Sweden [13].

The ever-increasing complexity in sample geometries from modern processing routes like additive manufacturing or investment casting drives the demand for highly flexible sample positioning. Simultaneously, the demand for high accuracy must be satisfied to perform residual stress or local texture analysis, as it is mandatory for both to measure at the exact same sample location from different directions. For this purpose, industrial robot systems with six degrees of freedom (6DoF) are a promising concept to solve this and further issues provided the position accuracy can be improved significantly. However, as mentioned earlier, the accuracy of robot systems is currently not able to match the necessary requirements from ISO standard 21432:2019 [14] for neutron residual stress determination and current benchmarking studies [1].

The individually calibrated robotic arm RX160 from Staubli at STRESS-SPEC possesses an absolute position accuracy of up to 500 μm with a quoted repeatability of 50 μm. This specification misses the required absolute positioning accuracy of 50 μm (defined as 10% of the smallest usefully achievable gauge volume). In addition, the widespread and unique measurement tasks have to be considered—sample weights vary from grams to many kilograms, influencing the accuracy of the system and preventing an in-advance calibration, which is possible for industrial processes in which the robot repeats the same process millions of times.

Below, an overview of the entire high accuracy positioning process route is given. The design aspects of the setup are described, with a focus on the optical system. Subsequently, the algorithm is explained in more detail regarding the optical tracking. After that, the motion correction algorithm, which turns visual feedback into motion correction commands for the robot, is presented. Finally, the visual servoing system is evaluated using a real sample and a 6DoF laser tracker.

## 2. Materials and Methods

### 2.1. Measurement Process Chain

**Figure 1:**
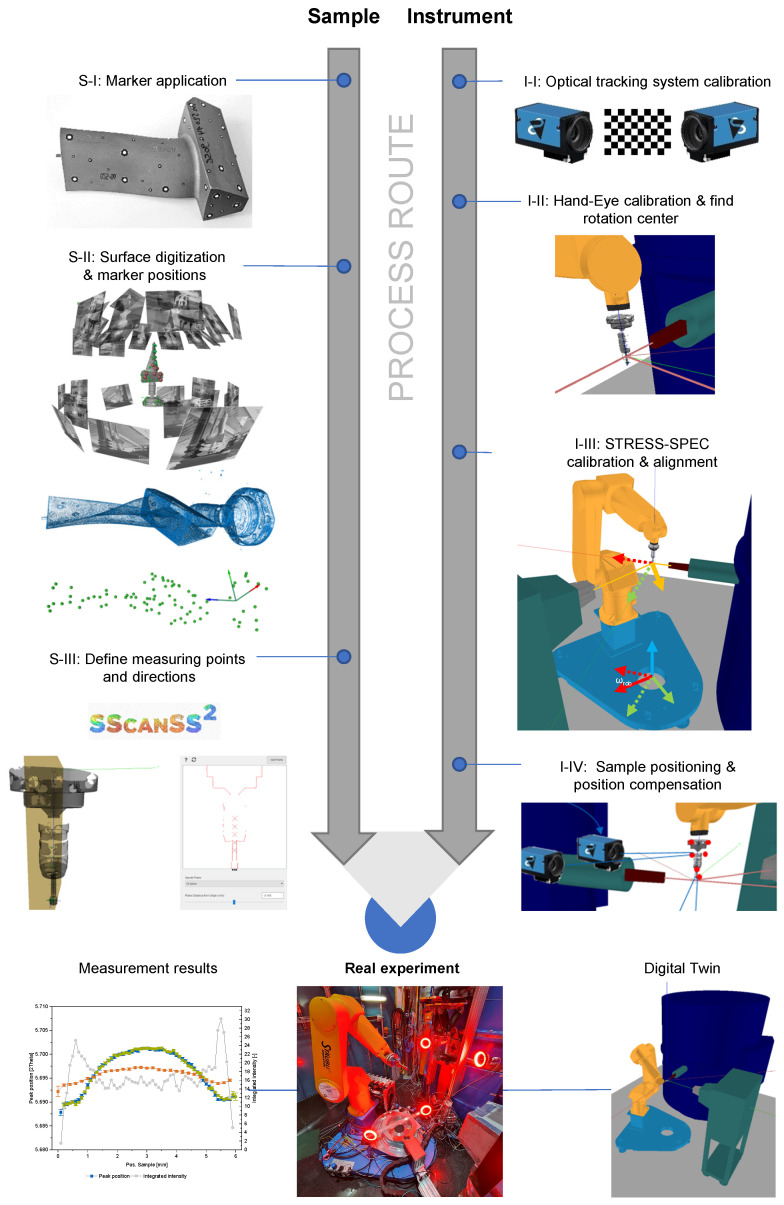
The entire process route consists of two branches, one for the sample and one for the instrument, most of which can be executed in parallel. The sample branch creates a digital version of the sample, mainly to obtain the marker positions and the measuring instructions. The instrument branch calibrates the optical system, the robot, and the diffractometer.

Figure 1 shows the main process chain of the robot positioning system, which is divided into the sample and instrument branches. The sample branch describes the steps to be finally able to define the measurement task (points and directions) and track the sample at the instrument. By following the adjacent instrument branch, the STRESS-SPEC diffractometer is prepared for the upcoming measurement procedure. Both sample and instrument preparation are finally merged in the procurement of the actual experiment.

At first, a dust- and oil-free sample surface must be provided for optical marker application (Step S-I). Concentric contrasting circle (CCC) markers [15], available in different sizes, are attached to the surface. Shiny surfaces are hard to scan in the surface digitization process. In this case, a temporary or persistent coating spray provides dulled reflection behavior. In addition to markers applied to the sample itself, sample holders with fixed markers are used to simplify the handling of different sample geometries and mounting on the robot flange. The appropriate choice of a sample coordinate system can then already be predefined by the sample holder.

After sample preparation, the marker positions and the sample surface are captured with a structured light scanner “CompactScan” from Carl Zeiss GOM Metrology GmbH (Braunschweig, Germany) (Step S-II). The marker positions from this measurement represent the reference pointset P, which is used later for position tracking. The scanned surface deviates from the original by a maximum of 15 µm according to the manufacturer. For the marker positions, even lower deviations are achieved. The digital sample model now allows the measurement points and directions to be defined/constructed virtually, which is beneficial for complex geometries (Step S-III). This can be performed via the open-source tool SScanSS 2 [16,17] or any other computer-aided design (CAD) tool available to the user. By using CAD tools, the surface scan can be enriched with the construction data of the parts to obtain information on inner structures. Where no CAD data are available, the data of a computer tomography (CT) scan can be used to provide this information.

Finally, the measurement task data, surface model, and marker positions in the sample coordinate system are exported and provided in a standard ASCII file format for the experiment at the diffractometer.

The camera system must be calibrated (Step I-I) before strain or texture analysis can be performed with the compensated robot positioning unit. Calibration is divided into two parts. The first is intrinsic and compensates for digital imaging errors such as lens distortion. As long as the lens and camera setup stays the same, this calibration must be performed only once. The second is an extrinsic calibration of the system, which describes how the individual cameras are oriented with respect to each other. This knowledge is essential for the triangulation of markers performed later during pose estimation.

The instrument also must undergo preparation for the experiment. This comprises the standard procedures for neutron beam alignment but also specifically to adjust the beam center on the rotation axis of the detector unit (Steps I-II). After this is carried out, the tracking system needs to be calibrated to this point in space to later match the gauge volume center with the desired measurement location (Steps I-III). Therefore, a reference sample is used, which has both features that can be tracked by the optical system and those that allow a precise localization of the neutron gauge volume. During Steps I-IV, the position of the sample is monitored as described in Section 2.3.1.

### 2.2. Design Aspects of the System

**Figure 2:**
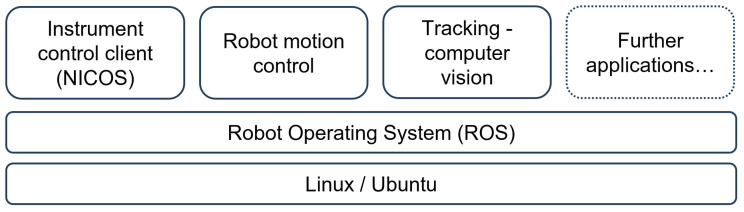
The digital twin rests on the two integration layers Linux (Ubuntu) and Robot Operating System (ROS). It consists of an easily extendable modular structure.

For instrument control, a data representation of all motors/actuators and sensors is available within the instrument control system NICOS [18], but without a link to a virtual geometrical representation. Such a geometrical representation is crucial for the automatic path planning of the robotic arm to avoid collisions. The integration layers of the digital twin are introduced in Figure 2. As a basic concept, the whole diffractometer, including all its movable parts, is modeled as a robot and implemented with the Robot Operating System (ROS) [19].

The utilized version is ROS 1 Noetic. The structure consists of a set of individual nodes, which communicate via messages with each other to exchange data and trigger specific functions. This modular architecture makes the system easier to maintain. Specific modules, e.g., the tracking module, can be easily updated later when better algorithms are available without changing the other parts of the architecture. The system also becomes more transparent, which facilitates troubleshooting in the case of failing components.

#### 2.2.1. Multi-Camera Setup

**Figure 3:**
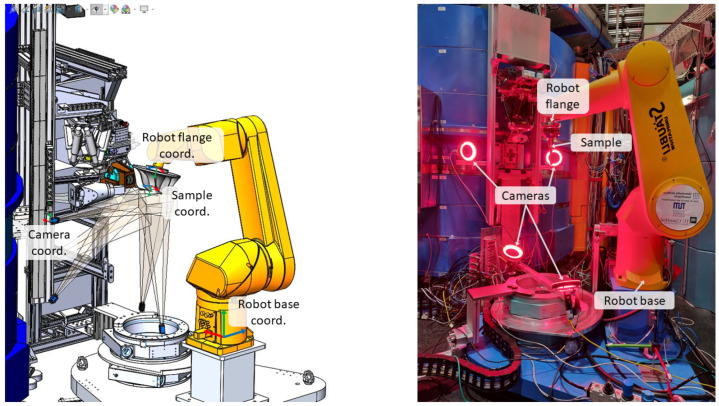
The planned multi-camera setup at STRESS-SPEC consists of six cameras, whereby a camera pair looks from each of three mutually perpendicular directions in order to increase the robustness of the pose estimation.

The main requirements for the multi-camera system are to cover the workspace well enough and with the desired accuracy so that the sample can be localized precisely and robustly. The camera system parameters and the process constraints influence these requirements. The number of cameras, the resolution and the color range of the camera sensors, the focal length of the optics, and the shutter type are the most important parameters of the camera system. The most relevant process parameters are the sample motion and the sample and workspace geometry. Our multi-camera setup, which fulfills these requirements, is able to cover a workspace of size 250×250×250 mm and can be seen in Figure 3.

The coverage needs to take into account sample and workspace geometry, occlusion by the robot and other components in the workspace, the number of cameras, and the poses of these cameras. The sample geometry determines which viewpoints it can be localized from accurately. For example, the features of a flat metal sheet can be detected more easily from the flat front than from its thin sides. The workspace geometry defines where the cameras can be mounted, within which space the sample moves, and which viewpoints are occluded by obstacles in the line of sight between this viewpoint and the sample. Feasible mounting spots for the cameras are restricted in our workspace due to the mechanical design of the STRESS-SPEC instrument environment. This limits the number of cameras that can be deployed. The final installment at STRESS-SPEC will be six cameras positioned at different positions, as indicated in Figure 3. One stereoscopic camera pair from each orthogonal direction is oriented towards the workspace center, i.e., the neutron gauge volume center. This enables us to cover as many positioning cases as possible within the workspace limits.

To enable the pose estimation algorithm, and thus the robot position algorithm, to reach the desired positioning accuracy of 50 μm or better, the data provided by the multi-camera system has to be fine-grained enough to allow this accuracy. The workspace resolution is the part of the workspace that is covered by one camera sensor pixel. It is determined by the pixel size on the camera sensor chip, the focal length of the lens and the distance between the camera and the workspace point.

**Figure 4:**
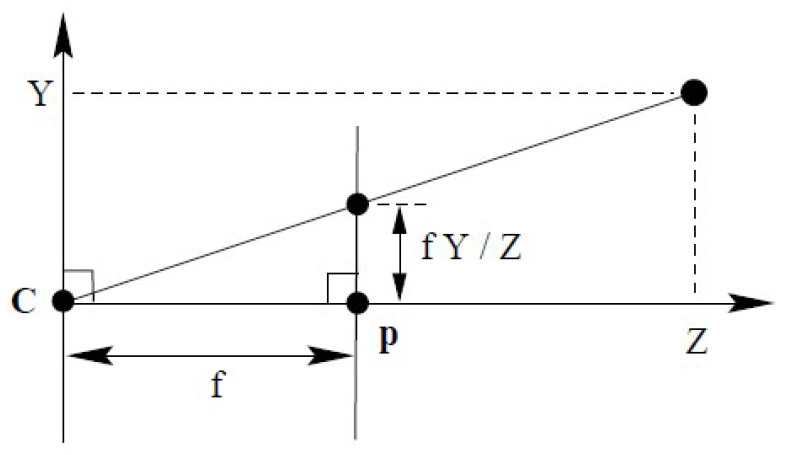
Pinhole camera model (adapted from [20]).

To determine the necessary camera resolution, i.e., the pixel size, number of pixels, and the focal length of the attached optics, the pinhole camera model was utilized, as shown in Figure 4 and described by Equation (1) [20]. A workspace resolution of less than $x_{world}=50\;\mu$m with cameras d=1 m away can be achieved with pixels of size $x_{cam}=2.4\;\mu$m at a focal length of f=50 mm. According to the Nyquist–Shannon theorem [21], this is not enough because the sampling rate has to be at least twice as high as the measured signal. In our case, this means that we can only measure features of size 100 μm. However, by utilizing sub-pixel approaches, values below the 50 μm range are achieved. To cover a large enough area in the workspace, we use a 20-megapixel (5472 × 3648) camera from The Imaging Source Europe GmbH (Bremen, Germany), which covers an area of 263 × 175 mm in our setup.

(1) $$x_{world}=\frac{x_{cam}}{f}*d$$

Gray-scale cameras offer better image resolution than color cameras for the same number of pixels because one pixel in a color image is generated by combining the intensity values of multiple sensor cells, which have different color filters on top of them. The interpolating effect of the Bayer filter in color cameras reduces the contrast and the accuracy of the pixel values. On the other hand, in gray-scale cameras, one image pixel corresponds to just one sensor cell, leading to sharper feature boundaries in the images.

In rolling shutter cameras, the image sensor is read line by line instead of all pixels at once, like in global shutter cameras. The rolling shutter reading method can be critical, especially for capturing moving objects in the scene. After the critical assessment and image evaluation of our use case, a non-moving sample assumption is considered valid while the images for its pose estimation are acquired (note: a neutron measurement at a specific point takes at least 10 sec while the sample is not moved). Therefore, rolling shutter cameras, which are considerably cheaper than global shutter cameras, are a suitable choice.

#### 2.2.2. Fiducial Marker System

Unique markers, like Aruco, would represent the ideal choice for the fiducial marker system, as already shown in previous work by [22]. Here, a single marker would already allow us to derive the pose and further markers would only be needed for redundancy. But they need a large enough planar surface on the sample in order to work. Curved sample surfaces are not suitable for those kinds of markers. In advance of the digitization process of the sample with the structured light scanner, CCC markers have been applied to the sample, which will also serve as fiducial markers for the optical tracking system at STRESS-SPEC.

A main advantage of this kind of marker is the invariant position of its midpoint, even if the marker is tilted to the camera plane. The size of a marker is important for small complex samples that have structures with a strong curvature. Here, the CCC marker type is beneficial as the midpoint can still be found with very high accuracy even if the marker diameter is below 1 mm. The blob detection algorithm of the OpenCV library provides a simple but suitable method to detect the CCC markers [23]. However, since the problem of identifying a single marker is not unique, it must be solved.

### 2.3. Point Set Registration

To derive the sample pose in space, the two pointsets (initial dataset from the 3D scanner and a subset from camera measurement) have to be brought to overlap or registered with respect to each other. The popular Iterative Closest Point (ICP) algorithm [24] did not lead to robust registration results after global registration. The two pointsets are small, and we did not find an accurate enough global registration method to prevent the ICP algorithm from sometimes finding only a local minimum of the optimization problem. After a first initial alignment, the ICP algorithm iteratively combines a search for nearest neighbors with a singular value decomposition (SVD) to derive rotation and translation between the pointsets. Since the nearest neighbor approach of the ICP algorithm did not provide reliable results, we implemented our own algorithm with the aim of identifying the corresponding points unambiguously in advance. This allows us in the subsequent step to directly use the SVD in the Kabsch algorithm [25] to derive translation and rotation between the two pointsets.

**Figure 5:**
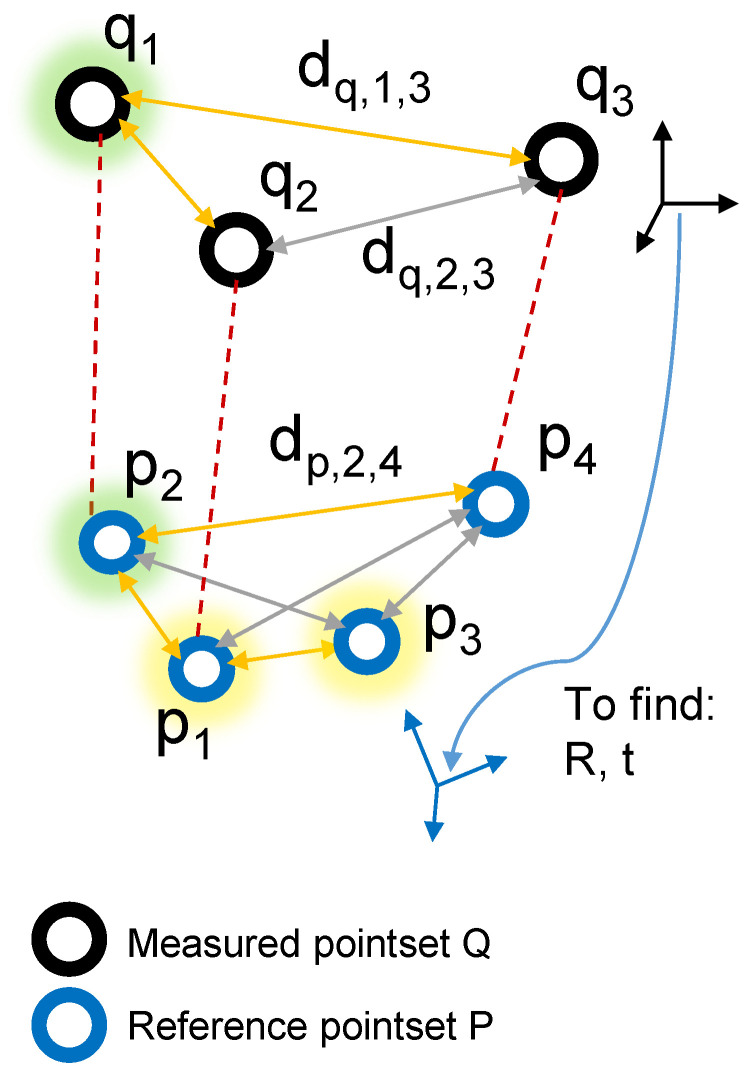
Comparison of the captured reference point set *P*, with the point set *Q* measured with the OTS. The corresponding point in *P* to point $q_{1}$ is looked for—the correct solution for q1 would be p2. Yellow marked points (p1 and p3) are candidate points, and yellow edges indicate common distances.

A unique feature is introduced to overcome the challenge of unambiguously identifying corresponding CCC markers in the reference and measured point set. A suitable unique feature of one CCC marker is the distance to all other markers in the observed point set. The task is to identify the corresponding points through a comparison of that feature (Figure 5) and finally to calculate the transformation (translation and rotation) between them. The algorithm is described schematically below and in more detail in Appendix A.

Input: The point set registration algorithm takes three inputs: the reference point set *P*, the measured point set *Q*, and the maximum accepted deviation σ of Euclidean point distances. *P* and *Q* are matrices where each row represents a 3D point, and σ determines the tolerance for matching distances.

Output: The algorithm outputs a rotation matrix *R* and a translation vector *t* representing the rigid transformation from the camera coordinate system to the sample coordinate system.

Compute Distances: The Euclidean distances between all points within the respective point sets *P* and *Q* are calculated.

Identify Candidate Points: The points from the measured point set *Q* are matched to points from the reference point set *P* based on their distances to the other points within their respective point set. To save computation time, this process is split into two steps. In the first step, only one distance is used to determine if the involved points could be a match. These points are called “candidate points”. After this first step, every point in *Q* has a list of candidate points, one of which is the true corresponding point.

Find Matches with Candidate Points: In the second step, all distances of the points in *Q* are compared with the distances of the points in their candidate lists. The candidate point with the most common distances corresponds to the final match.

After the correspondences between the two point sets is found, the translation vector *t* and the rotation matrix *R* representing the rigid transformation between them are computed using the Kabsch algorithm described by [25]. Since the sample position in space is now known, position errors can be corrected.

#### 2.3.1. Motion Correction Algorithm

**Figure 6:**
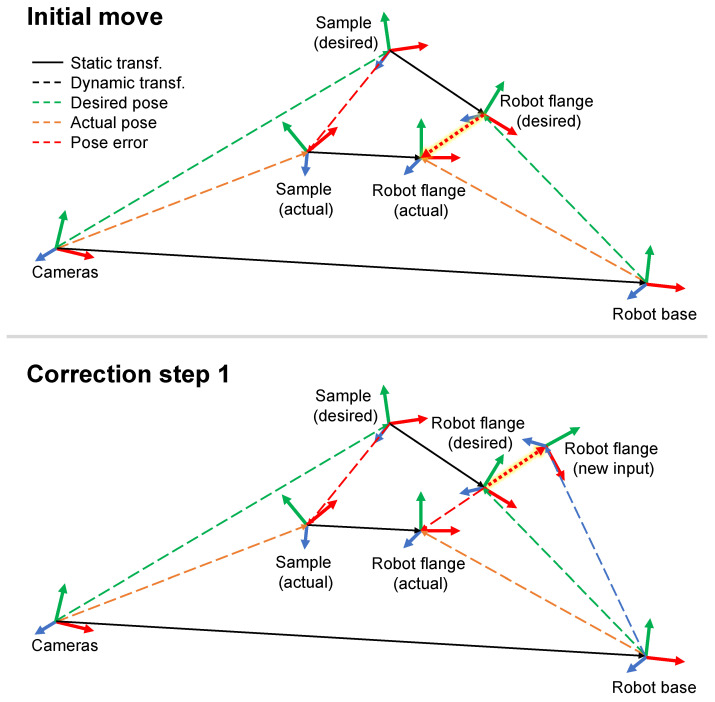
The robot motion error correction. The desired and actual state of the relevant coordinate systems and their transformations are shown. A straight line corresponds to a rigid body transformation that is static and does not change with the motion of the robot arm, while dotted lines indicate dynamic transformation behavior. Based on the deviation between the actual and desired sample position, the position deviation at the robot flange is calculated as a new robot movement input parameter for the first correction step.

The motion correction algorithm is based on an iterative deviation compensation method. An example of the principal idea can be found in [26]. The motion command to the robot is adjusted depending on the difference between the desired and actual pose of the sample, as visualized in Figure 6.

First, the desired sample pose relative to the camera system Tsample_desiredcameras is used to determine the desired robot flange pose relative to the robot base Tflange_desiredbase, which serves as the initial motion command for the robot controller, as shown in step 1 of Figure 6 and Equation (2).

(2) Tflange_desiredbase=Tcamerasbase·Tsample_desiredcameras·Tflange_desiredsample

As indicated in Figure 6 the two transformations Tcamerasbase and Tflange_desiredsample are rigid transformations which are derived using standard hand-eye calibration procedures [27].

After the initial move, the inaccuracies of the robot lead to a motion error. The deviation of the actual sample pose Tsample_actualcameras from the desired sample position is detected by the camera system. Based on this measurement, the actual flange pose Tflange_actualbase is determined. The difference between the desired and actual flange pose Tflange_actualflange_desired is the robot motion error (with respect to the desired sample position). To calculate the new corrected motion command to reach the desired sample position Tflange_newbase (Equation (3)), the opposite of the robot motion error is added onto the desired pose for correction (Figure 6, step 2).

(3) Tflange_newbase=Tflange_desiredbase·Tflange_actual−1flange_desired

These two steps, measuring the sample pose and correcting the motion error, are repeated until the sample reaches the desired pose within an acceptable deviation window. Even though the motion correction is based on the previous robot axis positions, the correction procedure still works, because the new position errors caused during the correction motion are small enough. The necessary correction steps are within the intrinsic absolute positioning accuracy of the robot (≈500 μm). Therefore, almost no change in the joint angles is needed to achieve the new position, and the correction of, e.g., the bending of joints is still valid.

## 3. Results

### 3.1. Experimental Setup

For proof of concept of the robot motion correction, a test setup close to a real measurement application was used. A welded plate with a size of 100×75×10 mm was attached to the robot flange and used as a pose-estimation reference object. This represents a typical sample geometry in a diffraction experiment. The procedure outlined in Figure 1 was applied to create the reference point cloud P of the sample. It was handled by a Stäubli TX2-60L robot and tracked by two 20-megapixel grayscale cameras of type DMK 33GX183 utilizing Fujinon CF50ZA-1S optics with 50 mm focal length. The sample was 1 m away from the baseline of the two cameras. The baseline of the camera setup was 1.3 m. The verification of the tracking system accuracy was performed with the 6DoF laser tracker API Radian R20 with an angular accuracy of 3.5 μm/m. The laser tracker was positioned 2 m in distance to the sample.

**Figure 7:**
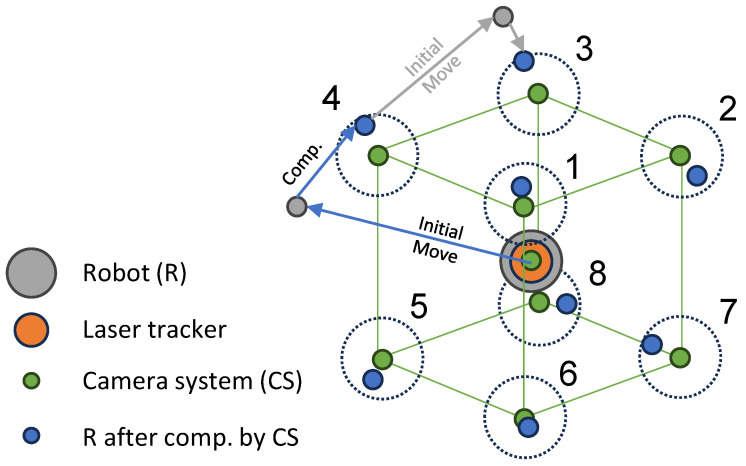
Testing positions in the cubes with various edge lengths. The measurement starts from Point 0 in the cube’s center point and continues in ascending order. The acceptance radius of 50 μm around each point is visualized, as is the initial robot movement and final position.

The robot positioning test was given by achieving the corner points of cubes of different sizes (10 mm, 20 mm, 30 mm, and 40 mm) in its working space according to ISO 9283. Figure 7 shows the order of approached points and the logic of compensation (as described in Figure 6) until a distance of 50 μm or better is reached. The evaluation starts at the center point of the the cube.

### 3.2. Robot Motion Compensation

**Figure 8:**
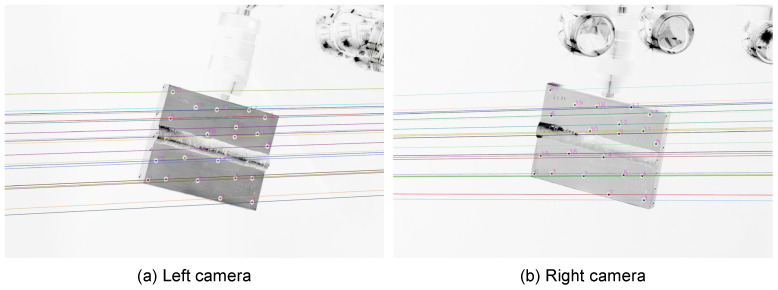
Camera images of a real welding sample, including epipolar lines and image-to-image matched point IDs. The sample size is 100×75×10 mm^3^.

Figure 8 shows the inverted images of the welded plate sample from both camera views. The colored lines correspond to the epipolar lines, which help to identify corresponding markers in stereoscopic camera setups [20]. Numbers are added as IDs of the CCC markers after identification as the same marker in both camera images. Out of these images, the measured pointset Q (Figure 5) is determined and used as described earlier for pose estimation.

**Figure 9:**
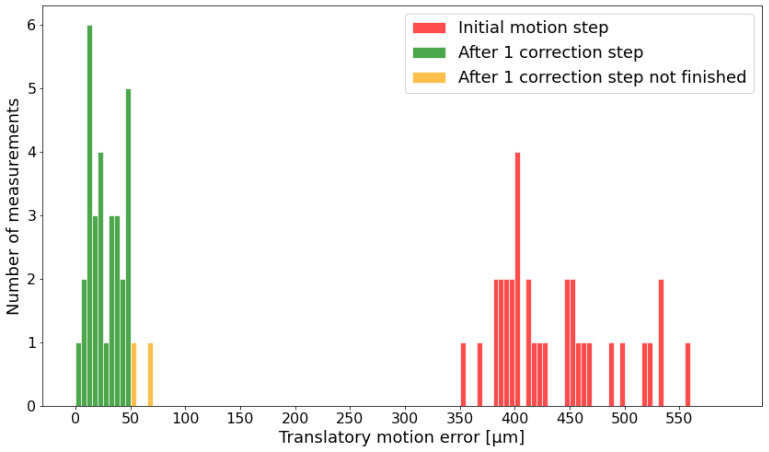
Translatory motion error after the initial motion and the further correction steps until the target is reached for all cube sizes, from 10 mm up to 40 mm, as determined by the camera system.

The task the robot has to achieve is to reach the corner points of a cube with sufficient accuracy. Four cube sizes (10 mm, 20 mm, 30 mm and 40 mm) are evaluated. The histogram in Figure 9 shows the motion correction steps, whereby the robot tries to move toward the desired sample pose (a corner-point of the cube), which is defined in the camera coordinate system. The red bars represent the accuracy of the robot motion based on camera feedback after the initial motion step (compare Figure 7). In this step, there is no real error correction because the robot just moves toward a target goal first and then, in the subsequent motion steps, corrects the resulting motion error. The green and yellow bars show the motion accuracy after the first correction step. Except for the two yellow bars, which needed seven correction steps, all motions achieved the desired translatory accuracy of less than 50μm within one correction. If a certain position cannot be reached after five steps, the robot gets the command to go back to the initial move position and start the procedure again.

**Figure 10:**
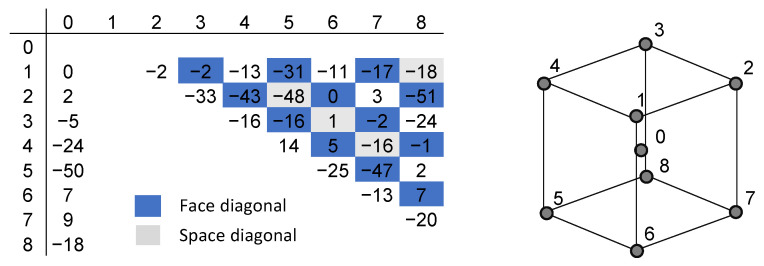
Distances between the measurement points are calculated out of the laser tracker data. The table contains the deviations of these distances to the lengths of a perfect cube with an edge length of 10 mm. The values are given in μm.

For the proof of concept of robot compensation, we will focus on the 10 mm cube and compare the positions achieved by the compensation with the laser tracker data. The goal was to reach the corner points (Points 1–8) from the cube center (Point 0). Distances were calculated between all points for the theoretically perfect cube (dataset 0), as measured by the camera system (dataset 1) and by the laser tracker (dataset 2). The table in Figure 10 compares the calculated distances of dataset 0 and dataset 2. Column “0” shows the evaluation from the center point to all corner points. The points were reached with an accuracy of 50μm or better. Checking the values for the face and space diagonals also confirms a well-shaped cube. Deviations of up to 100μm would be acceptable, as a worst-case scenario, if the length determining points are 50μm off in opposite directions.

**Figure 11:**
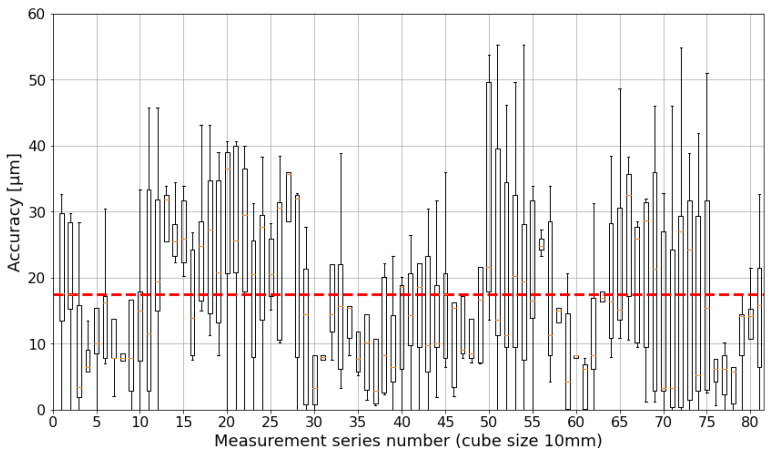
Absolute accuracy of translatory motion within a 10 mm cube. The boxplot is based on quartiles. The red horizontal line represents the mean value for all measurement values (≈18 μm).

**Figure 12:**
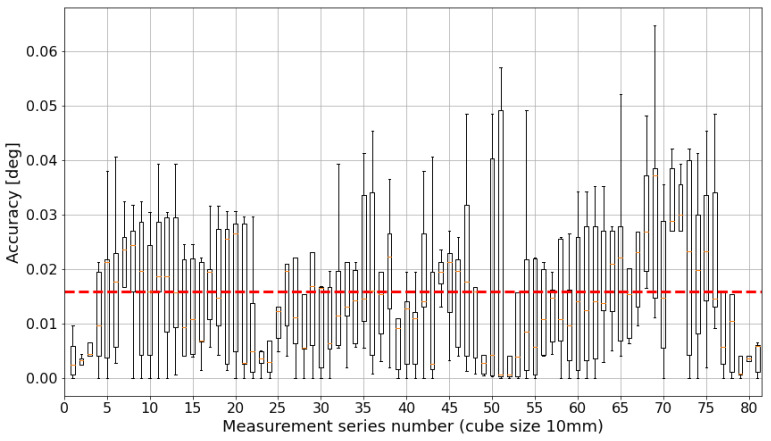
Absolute accuracy of rotatory motion within a 10 mm cube. The boxplot is based on quartiles. The red horizontal line represents the mean value for all measurement values (≈0.016°).

Further, the length estimation of the camera system is compared to the length estimation of the laser tracker (dataset 1 vs. dataset 2). The translatory motion accuracy in the 10 mm test field is shown in Figure 11 and mostly below the demanded threshold of 50μm. The rotatory motion error is 0.016° on average and also stays below the demanded threshold of 0.05° (Figure 12). It is less noisy than the translatory error.

**Figure 13:**
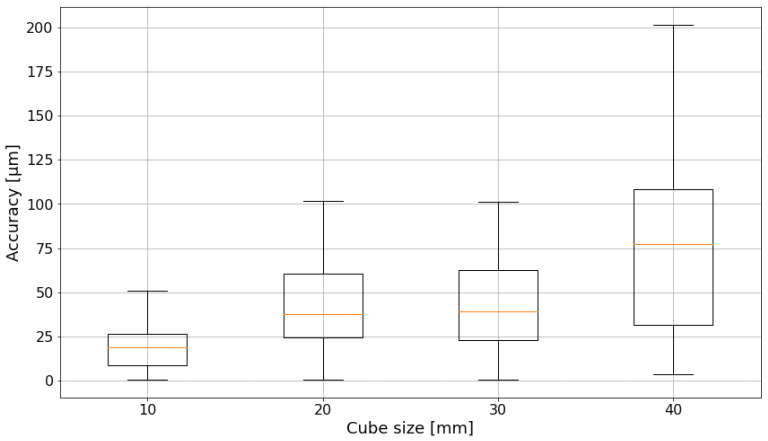
Absolute translatory accuracy of different cube sizes. The boxplot is based on quartiles. The orange line indicates the mean value.

In the overview of the translatory motion errors of all test field cube sizes in Figure 13, it can be seen that the absolute accuracy degrades with increasing cube sizes. Not only does the mean value get worse, but the noise levels also increase. Between cube size 10 mm and 20 mm, there is a considerable decrease in accuracy, but the values for the 20 mm and the 30 mm cubes differ only slightly. With the 40 mm cube, the results start to deteriorate, and the mean value even surpasses the desired 50μm threshold.

## 4. Discussion

The error in the absolute positioning of the robot depends on various factors, as already discussed in a previous work by [28]. The gears of the robot are not completely free of backlash, and the motors and encoders of the individual axes are subject to inaccuracies. The deflection of the individual axes is also a significant influencing factor. The deflection depends on the weight of the axis itself, the current position, and the attached sample weight. The thermal expansion depending on the temperature is also included in the inaccuracy, as are the manufacturing tolerances of the robot. The temperature level in the experimental hall of the FRM II is kept constant. The manufacturing- and machine-related inaccuracies have already been minimized as far as possible by calibration through the manufacturer according to ISO 9283, resulting in an absolute positioning accuracy of approx. 500μm. If a sample with large dimensions is measured, then deflection under its own weight can also occur here. The approach of positioning the reference markers as close as possible to the point to be measured counters all the previously mentioned variables influencing the accuracy. The correct positioning of the reference markers is all the more important. The previous observation suggests the positioning the reference markers in the vicinity of the measuring points. The identification of the individual markers for the determination of the pose is supported by an arrangement that is as random as possible. Symmetries lead to ambiguities. Also, if possible, all degrees of freedom should be well constrained. If, for example, all detected markers are arranged on a straight line in space, the rotational degree of freedom around this line is not restricted or only slightly restricted, and the measurement error of the tracking system increases accordingly.

**Figure 14:**
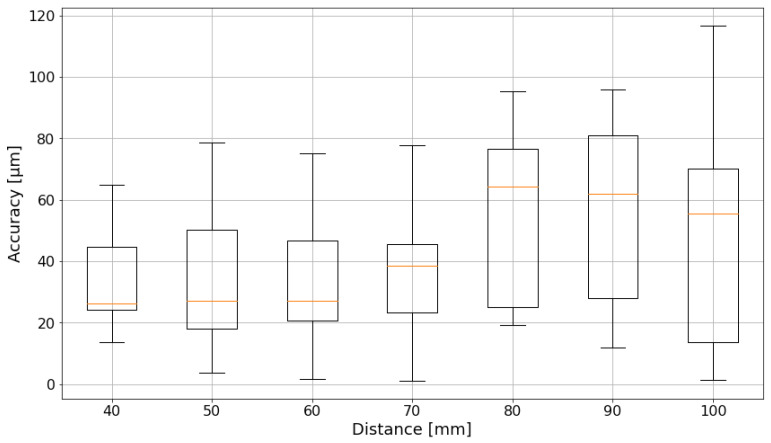
Absolute translatory accuracy of different square sizes parallel to the camera pair. The boxplot is based on quartiles. The orange lines indicate the mean value.

One of the main reasons why the accuracy degrades with increasing cube sizes is the cameras’ depth of field. The cameras are focused on a specific distance, at which they provide sharp images. The further away the sample is from this point, the blurrier the images get. To counter this effect, more view perspectives are necessary. Ideally, they should be perpendicular or vary enough in their pose to the other views in order to improve the pose estimation accuracy. The reason why only two cameras were used was to examine the functionality of the concept without adding additional complexity. Figure 14 shows the improved accuracy development for the movement along a square in a plane parallel to the baseline of the cameras. This gives much better results till the 70 mm edge length, but as the single camera still does not look perpendicular on the plane, the effect becomes dominant for larger movements again. A smaller focal length, e.g., 35 mm, will be tested in the future, as well as more intense lights, which allow a smaller aperture to be chosen.

A related effect that influences the accuracy of the tracking system is the angle between the camera axis (i.e., the axis perpendicular to the image plane) and the surface normal of the sample. When this angle gets bigger, then the feature gets distorted in the image; i.e., the circular fiducial marker on the sample becomes a slimmer ellipse in the image. This means that a larger part of the world is being mapped onto a single pixel. Thus, a certain feature detection error in the image leads to a bigger localization error in the workspace. A single camera would want to have a perpendicular view of the region of interest in the workspace to increase its workspace resolution. But for triangulation, which is used in order to determine the pose of the sample, the interplay of multiple camera views is important. The inaccuracies in detecting features on the image captured by the cameras result in uncertainties in the estimated position of a point in space. These uncertainties are reflected in the range of motion—that is, the set of possible positions—that this point can have within the field of view of the cameras due to the triangulation error resulting from the feature detection error. Multiple cameras looking perpendicularly at the same marker on the sample would result in poor accuracy in the depth direction. Therefore, the whole view topology, and not just individual views, needs to be considered when designing the camera setup.

The correction vector during the motion correction process is based on the measurements of the last step, i.e., the last static robot pose. But during the execution of the current correction step, new errors are introduced, which are not considered in the ongoing correction motion process. That could be the main reason why the motion correction sometimes needs seven steps to finish.

## 5. Conclusions

By walking through the steps of the sample and the instrument process route, the compensation of the robot positioning accuracy better than 50 μm was achieved. The therefore necessary design aspects of the optical tracking system, like camera models, lenses, and positions, were described in detail. The introduced concept includes the virtualization process of the sample and the instrument and the combination of their virtual copies in a digital twin supporting the real experiment. In particular, an algorithm for the point set registration was developed to track the sample position and implement the robot pose compensation.

After the feasibility of high position accuracy was shown, further enhancements in automation, user-friendly system handling, and portability of other instruments are addressed. Further research efforts in the domain of multi-camera view planning will prepare the ground for the optimal positioning of cameras tailored to the measurement task and workspace. On that basis, at least three stereoscopic camera pairs will be set up for high-accuracy positioning. In the first step, they will function redundantly and will not fuse their information in one data space. However, this will be rectified by a 3D camera calibration approach that creates one reference frame for all cameras. This will further significantly improve the tracking quality and stability of the system as more reference markers are used for point set registration and subsequent deviation compensation. The virtual representation of the sample and instrument holds the opportunity to implement the shortest neutron path through the sample strategy, which is derived automatically after the measurement task description. The shortest path saves measurement time by reducing neutron absorption within the sample and allows the use of the beamtime most efficiently. In addition, the digital twin holds the opportunity to provide offline user training, experiment definition, and simulation in advance.

## Data Availability

Dataset available on request from the authors.

## Abbreviations

    The following abbreviations are used in this manuscript:

6DoF	Six degrees of freedom
CCC	Concentric contrasting circle
CT	Computer tomography

## Appendix A

The algorithm described in Section 2.3 for point set registration, given in pseudocode.

**Algorithm 1** Point set registration algorithm
1: **Input:** reference point set $P\in R^{n\times 3}$; measured point set $Q\in R^{m\times 3}$; maximum accepted deviation σ of Euclidean point distances 2: **Output:** rotation matrix $R\in R^{3\times 3}$, translation vector $t\in R^{3}$ representing the rigid transformation from the camera coordinate system to the sample coordinate system 4: Compute distance matrices $D_{P}\in R^{n\times n}$, $D_{Q}\in R^{m\times m}$; $d_{p,k,l}\in D_{P}$ corresponds to the Euclidean distance between the points $p_{k}$, $p_{l}\in P$ and $d_{q,i,j}\in D_{Q}$ to the distance between $q_{i}$, $q_{j}\in Q$; 5: ▹ Identify candidate points that could be a match: 6: Initialize empty list $candidates[q_{i}]$ for candidate points from *P* that could be a match for the points $q_{i}$ from *Q*; only unique points, no double entries; 7: **for** each point $q_{i}$ in *Q* **do** 8: **for** each $d_{p,k,l}$ with k<l **do** 9: **if** $|d_{q,i,i+1}-d_{p,k,l}|<\sigma$ **then** 10: Add $p_{k},p_{l}$ to the list $candidates[q_{i}]$ 11: Add $p_{k},p_{l}$ to the list $candidates[q_{i+1}]$ 12: **end if** 13: **end for** 14: **end for** 15: ▹ Find all matches with candidate points: 16: $matches\left[q,p\right]\in R^{m\times n}$ contains number of matching edges for points q∈Q and p∈P 17: Initialize matches with zeros 18: **for** each $d_{q,i,j}$ **do** 19: **for** each $d_{p,k,l}$ with $p_{k}$ in $candidates[q_{i}]$ **do** 20: **if** $|d_{q,i,j}-d_{p,k,l}|\;<\sigma$ **then** 21: $matches\left[q_{i},p_{k}\right]=matches\left[q_{i},p_{k}\right]+1$ 22: Continue with next $p_{k}$ 23: **end if** 24: **end for** 25: **end for** 27: $q_{i}\in Q$ and $p_{k}\in P$ with the highest values in matches[q,p] are matched together 28: Get $R\in R^{3\times 3}$, $t\in R^{3}$ with Kabsch algorithm 29: Fine tuning: remove the point pair with the highest distance after registration and check if the overall registration quality improves 31: **return** R,t
